# Retreatment with anti‐PD‐1 antibody in non‐small cell lung cancer patients previously treated with anti‐PD‐L1 antibody

**DOI:** 10.1111/1759-7714.13241

**Published:** 2019-11-07

**Authors:** Kohei Fujita, Yuki Yamamoto, Osamu Kanai, Misato Okamura, Masayuki Hashimoto, Koichi Nakatani, Satoru Sawai, Tadashi Mio

**Affiliations:** ^1^ Division of Respiratory Medicine, Center for Respiratory Diseases National Hospital Organization Kyoto Medical Center Kyoto Japan; ^2^ Department of Drug Discovery for Lung Diseases, Graduate School of Medicine Kyoto University Kyoto Japan; ^3^ Division of Thoracic Surgery, Center for Respiratory Diseases National Hospital Organization Kyoto Medical Center Kyoto Japan

**Keywords:** Anti‐PD‐1, anti‐PD‐L1, immune checkpoint inhibitors, lung cancer, retreatment

## Abstract

**Background:**

This study evaluated the efficacy and safety of retreatment with anti‐programmed death 1 (anti‐PD‐1) antibodies in patients with advanced non‐small cell lung cancer (NSCLC) after prior treatment with anti‐programmed death‐ligand 1 (anti‐PD‐L1) antibodies.

**Methods:**

Data (*N* = 15) on patients' characteristics, number of cycles, regimens, their best response and immune‐related adverse events (irAEs) were recorded retrospectively.

**Results:**

NSCLC was initially treated with anti‐PD‐L1 antibody atezolizumab (*N* = 14) or durvalumab (*N* = 1). No patients had a high (≥50%) tumor expression of PD‐L1. The median cycles for atezolizumab were five (range 1–15), and median progression‐free survival was 2.8 and 6.0 months for atezolizumab and durvalumab, respectively. Five (33.3%) and nine (60.0%) patients showed stable and progressive disease as their best response, respectively. No differences in irAEs between anti‐PD‐L1 and anti‐PD‐1 antibodies occurred.

**Conclusion:**

Patients treated with anti‐PD‐L1 antibodies for NSCLC received limited benefits from retreatment with anti‐PD‐1 antibodies.

## Introduction

Several large‐scale clinical trials and clinical experiences have established the remarkable benefits of immune checkpoint inhibitors (ICI); anti‐programmed death‐1 (PD‐1) and PD‐ligand 1 (PD‐L1) in the treatment of patients with non‐small cell lung cancer (NSCLC).^1^, ^2^, ^3^, ^4^, ^5^ Thus, cancer immunotherapy continues to receive attention in research.

ICIs are increasingly used in the real‐world clinical setting, leading to questions on retreatment with ICIs. Very few studies report the efficacy and safety of ICI retreatment in patients with melanoma and NSCLC.^6^, ^7^, ^8^, ^9^, ^10^, ^11^ These studies show limited benefits with ICI retreatment. Even though ICIs are an acceptable option for the elderly or frail patients, the high costs associated with the treatment poses a heavy economic burden.^12^ The selection of appropriate candidates for ICI retreatment is therefore important. It is also imperative to accumulate data on retreatment with various ICIs.

We have previously published studies on both anti‐PD‐1 antibody and anti‐PD‐L1 antibody retreatment after treatment with anti‐PD‐1 antibody.^8^, ^10^ Until now, no study has evaluated the subsequent treatment with the anti‐PD‐1 antibody after the initial treatment with the anti‐PD‐L1 antibody. Therefore, this study aimed to evaluate the efficacy and safety of retreatment with anti‐PD‐1 antibody after NSCLC treatment with anti‐PD‐L1 antibody.

## Methods

This retrospective cohort study was conducted at the National Hospital Organization Kyoto Medical Center, Kyoto, Japan. We reviewed NSCLC patients who received anti‐PD‐1 antibodies after anti‐PD‐L1 antibodies between January 2018 and August 2019. At the time of this study, nivolumab and pembrolizumab were administered as anti‐PD‐1 antibodies, while atezolizumab and durvalumab were the anti‐PD‐L1 antibodies used. The inclusion criteria were; (i) pathologically confirmed NSCLC, and (ii) retreatment with nivolumab or pembrolizumab monotherapy after treatment with prior atezolizumab or durvalumab monotherapy. We excluded those who received combination therapy of cytotoxic agents and ICIs.

The data collected were; patients characteristics, number of treatment cycles, progression‐free survival (PFS) in patients treated with anti‐PD‐1 and anti‐PD‐L1 antibodies, treatment regimens for both anti‐PD‐L1 and anti‐PD‐1 antibodies, best response, and immune‐related adverse events (irAEs). Treatment response was evaluated based on the Response Evaluation Criteria in Solid Tumors version 1.1, and irAEs were evaluated based on the Common Terminology Criteria for Adverse Events version 4.0. This study protocol was approved by the Ethical Committee and the Institutional Review Board of National Hospital Organization Kyoto Medical Center (approved number: 019–044).

## Results

### Patients’ characteristics

A total of 15 patients were analyzed for this study. Table 1 shows the characteristics of the study patients. Fourteen patients received atezolizumab, and one received durvalumab as the initial anti‐PD‐L1 antibody. Since the patient who received durvalumab was eligible for concurrent chemoradiotherapy, durvalumab was also used for maintenance therapy. The mean age at induction of the initial anti‐PD‐L1 antibody was 71.4 ± 6.8 years, and all but one patient were male. Seven patients had adenocarcinoma of which one patient harbored the mutation for epidermal growth factor receptor. None had high (≥50%) tumor PD‐L1 expression at the time of diagnosis.

**Table 1:** Patients’ characteristics

Patients’ characteristics	*n* = 15
Age, years	71.4 ± 6.8
Sex (female/male)	1/14
Smoking history, n (%)	14 (93.3)
Body Mass Index, mean ± SD	21.8 ± 3.0
Histopathology	
Adenocarcinoma, n (%)	7 (46.7)
Squamous carcinoma, n (%)	6 (40.0)
NOS, n (%)	2 (13.3)
Performance status (2≤), n (%)	2 (13.3)
Driver mutations	
EGFR mutation, n (%)	1 (6.7)
PD‐L1 expression	
TPS ≤50%, n (%)	0 (0.0)
1% ≤ TPS < 50%, n (%)	5 (33.3)
TPS <1%, n (%)	5 (33.3)
Unknown, n (%)	5 (33.3)
Clinical Staging	
stage 3A/B/C, n (%)	10 (66.7)
stage 4A/B, n (%)	4 (26.7)
Postoperative recurrence, n (%)	1 (6.7)
Initial anti‐PD‐L1 antibody	
Atezolizumab, n (%)	14 (93.3)
Durvalumab, n (%)	1 (6.7)

EGFR, epidermal growth factor receptor; NOS, not otherwise specified; PD‐L1, programmed death‐ligand 1; TPS, tumor proportion score.

### Initial treatment with anti‐PD‐L1 antibody

Table 2 shows the initial treatment profile with the anti‐PD‐L1 antibody. The median cycle of atezolizumab therapy was five (range = 1–15). Only 4/14 (28.6) patients maintained stable disease (SD) and no patient achieved partial or complete response with atezolizumab. All patients treated with atezolizumab had prior cytotoxic chemotherapy, of which three patients received ICIs (anti‐PD‐1 antibodies) before initial atezolizumab. These patients received different anti‐PD‐1 antibodies before and after atezolizumab treatment. The median PFS was 2.8 (range 0.6–10.3) months in patients with atezolizumab and 6.0 months with durvalumab. The patient treated with durvalumab received concurrent chemoradiotherapy as the first‐line treatment, and maintenance therapy was deemed a failure at sixth months. All patients discontinued the initial anti‐PD‐L1 antibodies due to disease progression.

**Table 2:** Treatment profiles of initial anti‐PD‐L1 antibody

Initial anti‐PD‐L1 antibody	Atezolizumab	Durvalumab
	*n* = 14	*n* = 1
Median cycle length, months (range)	5 (1–15)	14
PD‐L1 expression		
TPS ≥50%, n (%)	0 (0.0)	0
1% ≤ TPS < 50%, n (%)	4 (28.6)	1
TPS <1%, n (%)	5 (35.7)	0
NE, n (%)	5 (35.7)	0
PFS, months (range)	2.8 (0.60–10.3)	6.0
Best response during anti‐PD‐L1 antibody treatment		
PD, n (%)	9 (64.3)	0
SD, n (%)	4 (28.6)	1
NE, n (%)	1 (7.1)	0
Treatment prior to anti‐PD‐L1 antibody		
Cytotoxic chemotherapy		
CBDCA+nabPTX/PTX ± BV, n (%)	7 (50.0)	1
CBDCA+PEM ± BV, n (%)	7 (50.0)	0
DTX + RAM, n (%)	2 (14.3)	0
Immune checkpoint inhibitors, n (%)	3 (21.4)	0
Others, n (%)	5 (35.7)	0
Radiotherapy (60Gy), n (%)	0 (0.0)	1

BV, bevacizumab; CBDCA, carboplatin; DTX, docetaxel; nabPTX, nanoparticle albumin‐bound paclitaxel; NE, not evaluated; PD, progressive disease; PD‐L1, programmed death‐ligand 1; PEM, pemetrexed; PFS, progression‐free survival; RAM, ramucirumab; SD, stable disease; TPS, tumor proportion score.

### Subsequent treatment with anti‐PD‐1 antibody

Table 3 shows the treatment profiles of subsequent anti‐PD‐1 antibody treatment with nivolumab (*N* = 7) and pembrolizumab (*N* = 8). The median cycles of nivolumab and pembrolizumab were four (range = 1–7) and four (range = 1–14), respectively. Five (71.4%) patients showed progressive disease (PD), and one (14.3%) showed SD as their best response for nivolumab retreatment. Four (50.0%) patients showed PD and three (37.5%) patients showed SD as best response for pembrolizumab retreatment. None showed partial or complete response. The median PFS was 1.9 (range 0.4–3.0) months with nivolumab and 2.8 (range 0.47–13.4) with pembrolizumab.

**Table 3:** Treatment profiles of anti‐PD‐1 antibody retreatment

Anti‐PD‐1 antibody retreatment	Nivolumab	Pembrolizumab
	*n* = 7	*n* = 8
Median cycle length, months (range)	4 (1–7)	4 (1–14)
PD‐L1 expression		
TPS ≤50%, n (%)	0 (0.0)	0 (0.0)
1% ≤ TPS < 50%, n (%)	1 (14.3)	4 (50.0)
TPS <1%, n (%)	4 (57.1)	1 (12.5)
NE, n (%)	2 (28.6)	3 (37.5)
PFS, months (range)	1.9 (0.43–3.0)	2.8 (0.47–13.4)
Best response during anti‐PD‐1 antibody treatment		
PD, n (%)	5 (71.4)	4 (50.0)
SD, n (%)	1 (14.3)	3 (37.5)
NE, n (%)	1 (14.3)	1 (12.5)
Treatment between anti‐PD‐L1 antibody and anti‐PD‐1 antibody		
Cytotoxic chemotherapy		
CBDCA+nabPTX/PTX ± BV, n (%)	1 (14.3)	0 (0.0)
CBDCA+PEM ± BV, n (%)	0 (0.0)	0 (0.0)
DTX + RAM, n (%)	3 (42.9)	1 (12.5)
Others, n (%)	0 (0.0)	2 (25.0)

anti‐PD‐1, anti‐programmed death 1; BV, bevacizumab; CBDCA, carboplatin; DTX, docetaxel; nabPTX, nanoparticle albumin‐bound paclitaxel; NE, not evaluated; PD, progressive disease; PD‐L1, programmed death‐ligand 1; PEM, pemetrexed; PFS, progression‐free survival; RAM, ramucirumab; SD, stable disease; TPS, tumor proportion score.

Although the overall results of anti‐PD‐1 antibodies retreatment showed poor response, the number of patients with SD as best response and the median PFS was slight higher for pembrolizumab retreatment compared to nivolumab retreatment.

### Immune‐related adverse events

The occurrences of irAEs are shown in Table 4. Although skin rash and fever were the frequently observed irAEs with both initial anti‐PD‐L1 antibody and subsequent anti‐PD‐1 antibody treatment, no patient experienced severe irAEs. Two patients had grade 3 interstitial pneumonia and grade 3 bacterial pneumonia after induction with anti‐PD‐1 antibody. These patients fully recovered with adequate treatment.

**Table 4:** Profiles of immune‐related adverse events

Immune‐related adverse event	Initial anti‐PD‐L1 antibody		Subsequent anti‐PD‐1 antibody	
	G1	≥G2	G1	≥G2
Rash	3	5	3	1
Infection	0	0	0	2
Elevation of liver enzyme	1	0	0	1
Fatigue	0	3	0	1
Interstitial pneumonia	0	1	0	2
Fever	2	4	3	2
Hypothyroidism	0	1	0	0

All values are represented as n. anti‐PD‐1, anti‐programmed death 1; G, grade according to the Common Terminology Criteria for Adverse Events version 4.0; PD‐L1, programmed death‐ligand 1.

## Discussion

This study shows poor response of NSCLC to anti‐PD‐1 antibody retreatment (nivolumab/pembrolizumab) after initial treatment with anti‐PD‐L1 antibodies (atezolizumab/durvalumab). The study results are consistent with previous studies that show limited benefits with ICI retreatment.^6^, ^7^, ^8^, ^9^, ^10^, ^11^ However, certain factors positively predict the efficacy of ICI retreatment such as very high PD‐L1 expression (tumor proportion score, TPS ≥80%),^8^ favorable response to initial ICIs,^6^, ^7^ or radiotherapy before ICI retreatment.^11^ The fact that none of the patients presented with ≥50% TPS or a favorable response to initial anti‐PD‐L1 antibody treatment, could explain the poor response to subsequent anti‐PD‐1 antibodies in this study. Even with a small sample, pembrolizumab retreatment was slightly more effective than nivolumab retreatment. In our study participants, patients receiving pembrolizumab retreatment had higher proportion of positive PD‐L1 expression (1% ≤ TPS < 50%) than patients with nivolumab retreatment as shown in Table 3. This might be one of the reasons for the favorable results in the pembrolizumab retreatment. Also, in our cohort, three patients received anti‐PD‐1 antibody before initial anti‐PD‐L1 antibody, amounting to triple ICI treatment.

All patients in this study received the initial anti‐PD‐L1 antibodies as the second or later line regimen. Since this study evaluated the efficacy of anti‐PD‐1 antibodies after anti‐PD‐L1 treatment, we did not consider the treatment before anti‐PD‐L1 antibodies. Therefore, all patients in this study to some degree suffered from physical exhaustion and immune compromise. Lung cancer acquires resistance to immunotherapy with ICIs due to the loss of T cell function, lack of T cell recognition by downregulation of tumor antigen presentation, and development of escape mutation variants.^13^ Thus, the prolonged use of ICIs might exhaust the host immune status and contribute to the poor response to subsequent ICI treatments. The present study is in line with previous studies that show limited efficacy regardless of the type, sequence, and timing of ICI retreatment. Overall, the data suggest that retreatment with ICIs is a limited option for NSCLC.

There are several limitations to our study. This study was retrospective and conducted in a single hospital, with a small number of patients. There is possible selection bias, and the results must be interpreted with caution. The timing and selection of all regimens were determined by the attending doctors, and therefore not standardized between patients. Statistical analysis could not be performed due to the small sample size. However, our study is preliminary, and we recommend future prospective, multicenter, large sample studies with subgroup analyses to explore the results of this study.

In conclusion, retreatment of NSCLC with anti‐PD‐1 antibody after treatment with anti‐PD‐L1 antibody shows only limited benefits. The positive predictive factors determining retreatment outcomes must be carefully considered during patient selection for ICI rechallenge.

## Disclosure

Kohei Fujita has received honoraria from Boehringer Ingelheim. Tadashi Mio has received honoraria from Bristol Myers Squibb, Chugai Pharmaceutical, AstraZeneca, Novartis, and Boehringer Ingelheim. The other authors have no conflicts of interest to declare.
